# The protective effect of ischemic preconditioning on rat testis

**DOI:** 10.1186/1477-7827-5-47

**Published:** 2007-12-20

**Authors:** Tayfun Sahinkanat, K Ugur Ozkan, Fatma I Tolun, Harun Ciralik, Secil S Imrek

**Affiliations:** 1 Department of Urology, University of Kahramanmaras Sutcu Imam, School of Medicine, Kahramanaras, Turkey; 2 Department of Pediatric Surgery, University of Kahramanmaras Sutcu Imam, School of Medicine, Kahramanaras, Turkey; 3 Department of Biochemistry, University of Kahramanmaras Sutcu Imam, School of Medicine, Kahramanaras, Turkey; 4 Department of Pathology, University of Kahramanmaras Sutcu Imam, School of Medicine, Kahramanaras, Turkey

## Abstract

**Background:**

It has been demonstrated that brief episodes of sublethal ischemia-reperfusion, so-called ischemic preconditioning, provide powerful tissue protection in different tissues such as heart, brain, skeletal muscle, lung, liver, intestine, kidney, retina, and endothelial cells. Although a recent study has claimed that there are no protective effects of ischemic preconditioning in rat testis, the protective effects of ischemic preconditioning on testicular tissue have not been investigated adequately. The present study was thus planned to investigate whether ischemic preconditioning has a protective effect on testicular tissue.

**Methods:**

Rats were divided into seven groups that each contained seven rats. In group 1 (control group), only unilateral testicular ischemia was performed by creating a testicular torsion by a 720 degree clockwise rotation for 180 min. In group 2, group 3, group 4, group 5, group 6, and group 7, unilateral testicular ischemia was performed for 180 min following different periods of ischemic preconditioning. The ischemic preconditioning periods were as follows: 10 minutes of ischemia with 10 minutes of reperfusion in group 2; 20 minutes of ischemia with 10 minutes of reperfusion in group 3; 30 minutes of ischemia with 10 minutes of reperfusion in group 4; multiple preconditioning periods were used (3 × 10 min early phase transient ischemia with 10 min reperfusion in all episodes) in group 5; multiple preconditioning periods were used (5, 10, and 15 min early phase transient ischemia with 10 min reperfusion in all episodes) in group 6; and, multiple preconditioning periods were used (10, 20, and 30 min early phase transient ischemia with 10 min reperfusion in all episodes) in group 7. After the ischemic protocols were carried out, animals were sacrificed by cervical dislocation and testicular tissue samples were taken for biochemical measurements (protein, malondialdehyde, nitric oxide) and histological examination.

**Results:**

Although decreased tissue malondialdehyde levels were detected in the groups of 2, 3, 4, and 5 compared to group 1, significant decreases were observed in only group 2 and group 5 (p < .05). Nitric oxide levels were numerically decreased in all groups compared to the control group but was statistically significant only in group 5 (p < .05). Histopathological examination demonstrated that all groups subjected to ischemic preconditioning had less tissue damage than group 1 (p < .05).

**Conclusion:**

These results suggest that ischemic preconditioning provides tissue protection in testicular tissue.

## Introduction

Testicular torsion is an acute clinical condition in humans that must be treated promptly to avoid loss of the ipsilateral testis. Long-term follow-up has shown subsequent late atrophy of the affected testis. Testicular damage after spermatic cord torsion is related to the duration of ischemia and to the severity of the torsion [1,2]. The main pathophysiology of testicular torsion is ischemia-reperfusion injury of the testis caused by the twisted spermatic cord and its release [3,4], which is most likely mediated by oxygen free radicals [4].

It is well-known from studies of different tissues such as heart, brain, skeletal muscle, lung, liver, intestine, kidney, retina and endothelial cells [5-9] that brief episodes of sublethal ischemia-reperfusion, so called ischemic preconditioning, provide powerful tissue protection against ischemic tissue damage. The protective effect of ischemic preconditioning occurs in both early and delayed phases. Early preconditioning occurs within minutes, delayed preconditioning occurs 24 h after ischemic preconditioning and induces less protection.

A recent study [10] has claimed that there are no protective effects of ischemic preconditioning against subsequent ischemia on rat testis. But, the possible protective effects of ischemic preconditioning on testicular tissue should be investigated adequately with new different protocols because, in the clinical practice, the protective effect of ischemic preconditioning on the testis might be usefull in the first step of staged orchiopexy for abdominal testis.

The present study was planned to investigate whether ischemic preconditioning has a protective effect on subsequent testicular ischemic tissue injury by using biochemical parameters and histolological examination.

## Methods

Prepubertal, male, albino rats (180–200 g) were acquired from university vivarium sources and maintained on a 12 h light/12 h dark cycle with *ad libitum *food and water at 20–24°C. On the day of the experiment, animals were anesthetized with ketamine (50 mg/kg) and the surgical operation described below was performed. Our experimental research on animals followed internationally recognized guidelines. Ethic committee of our university gave approval to the study, with a reference number of 2007/01.

### Surgery

#### Ischemic preconditioning

After the induction of anesthesia, a left scrotal incision was made. The tunica vaginalis was opened, and the testicle was delivered to the surgical field. The testicle was rotated 720° in a clockwise direction and maintained in this torsion position by fixing the testicle to the scrotum with a 5–0 silk suture. At the end of testicular ischemia period the testicle was released for the perform reperfusion period.

#### Main ischemia

By the same incision and surgical procedure described above, the main ischemia period lasting 180 min was performed just following ischemic preconditioning periods. After all procedures were completed, the incision was closed and the animal was kept on a heating pad to maintain its body temperature. At the end of the procedures, the testes were removed and divided longitudinally into two halves for biochemical measurements (protein, malondialdehyde (MDA), nitric oxide (NO)) and histological examinations. Then all animals were sacrificed by cervical dislocation.

### Experimental protocol

Animals were divided into seven groups each containing seven rats. Each group underwent the procedure described below.

Group 1: No preconditioning + 180 min ischemia (control group)

Group 2: 10 min transient ischemia + 10 min reperfusion + 180 min ischemia

Group 3: 20 min transient ischemia + 10 min reperfusion + 180 min ischemia

Group 4: 30 min transient ischemia + 10 min reperfusion + 180 min ischemia

Group 5: Three consecutive "10 min transient ischemia + 10 min reperfusion" cycles + 180 min ischemia

Group 6: Three consecutive ''5, 10, and 20 min, respectively, transient ischemia + 10 min reperfusion" cycles + 180 min ischemia

Group 7: Three consecutive ''10, 20, and 30 min, respectively, transient ischemia + 10 min reperfusion" cycles + 180 min ischemia

### Biochemical analyses

The testes were homogenized separately in phosphate buffer and malondialdehyde (MDA) and nitric oxide (NO) were measured in the specimens. MDA detection was based on the measurements of the absorbance of thiobarbituric acid-malondialdehyde [11]. NO was measured with the Griess method by the detection of nitrite levels [12]. Protein was measured according to Lowry et al. [13].

### Histological examination

The testicles immersed in Bouin's fixative were dehydrated in alcohol and embedded in paraffin blocks, sectioned at 5 μm, and stained with hematoxylin and eosin. The light microscope histological examination of the slides prepared from middle portion of each hemi-testis was done by a pathologist in a blinded fashion. The 4-level grading scale of Cosentino et al. [14], was used to quantify histological injury. Grade 1 showed normal testicular architecture with an orderly arrangement of germinal cells. Grade 2 injury showed less orderly, noncohesive germinal cells, and closely packed seminiferous tubules. Grade 3 injury exhibited disordered, sloughed germinal cells with shrunken pyknotic nuclei and less distinct seminiferous tubule borders. Grade 4 injury defined seminiferous tubules that were closely packed with coagulative necrosis of the germinal cells.

### Statistical analysis

MDA and NO levels were expressed as nmol/mg protein and *μ*mol/mg protein, respectively. The results were given as sum or mean ± SD. Statistical analysis was done by Kruskall Wallis analysis of variance, Mann-Whitney U test and Fischer's exact Chi-square test where appropriate. The level of statistical significance was p < 0.05.

## Results

No animals died because of the procedure.

### MDA levels

The results of testicular MDA levels in all groups are shown in table 1. Although numerically decreased tissue MDA levels were detected in experimental groups of 2, 3, 4, and 5 compared to group 1, significant decreases were observed in only group 2 and group 5 (p < .05). Paradoxically MDA levels were numerically increased in the groups of 6 and 7 compared to the control group.

**Table 1:** MDA and NO levels in ipsilateral testes of rats after undergoing testicular ischemia with or without ischemic preconditioning. Data are expressed as mean ± SD. *p < 0.05 vs. Group 1.

**Group**	**Intervention**	**MDA **(nmol/mg protein)	**NO **(*μ*mol/mg protein)
**1**	No preconditioning + 180 min ischemia	0.22 ± 0.08	0.44 ± 0.24
**2**	"10 min transient ischemia + 10 min reperfusion" + 180 min ischemia	0.05 ± 0.03*	0.21 ± 0.20
**3**	"20 min transient ischemia + 10 min reperfusion" + 180 min ischemia	0.15 ± 0.09	0.29 ± 0.15
**4**	"30 min transient ischemia + 10 min reperfusion" + 180 min ischemia	0.17 ± 0.08	0.32 ± 0.15
**5**	"3 × consecutive 10 min transient ischemia + 10 min reperfusion" + 180 min ischemia	0.05 ± 0.03*	0.07 ± 0.04*
**6**	"3 consecutive 5 × 10 × 20 min transient ischemia + 10 min reperfusion" + 180 min ischemia	0.27 ± 0.10	0.42 ± 0.21
**7**	"3 consecutive 10 × 20 × 30 min transient ischemia + 10 min reperfusion" + 180 min ischemia	0.25 ± 0.15	0.34 ± 0.18

MDA: malondialdehyde; NO: nitric oxide

### NO levels

NO levels were numerically decreased in all groups compared to the control group but this was statistically significant in only group 5 (p < .05) (Table 1).

### Histopathology

**Figure 1:**
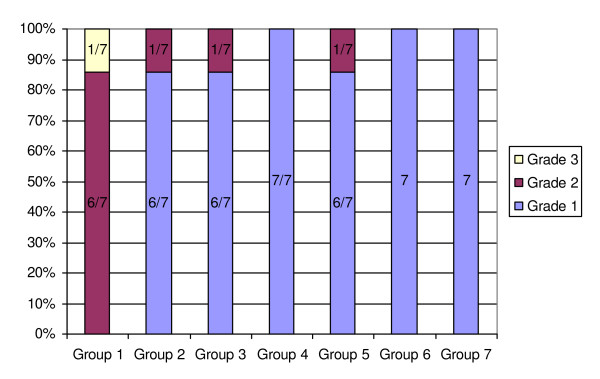
**Distribution of different grades of histopatological testicular injuries in all experimental groups**. Group 1: No preconditioning + 180 min ischemia (control group). Group 2: 10 min transient ischemia + 10 min reperfusion + 180 min ischemia. Group 3: 20 min transient ischemia + 10 min reperfusion + 180 min ischemia. Group 4: 30 min transient ischemia + 10 min reperfusion + 180 min ischemia. Group 5: Three consecutive "10 min transient ischemia + 10 min reperfusion" cycles + 180 min ischemia. Group 6: Three consecutive ''5, 10, and 20 min, respectively, transient ischemia + 10 min reperfusion" cycles + 180 min ischemia. Group 7: Three consecutive ''10, 20, and 30 min, respectively, transient ischemia + 10 min reperfusion" cycles + 180 min ischemia

**Figure 2:**
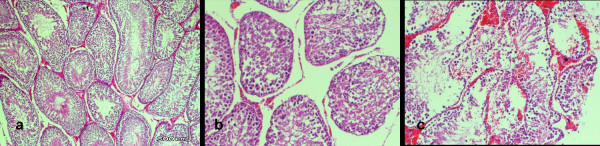
Photomicrographs of cross-sections of the different histopathological grades: a, grade 1; b, grade 2; c, grade 3. (H&E, a, ×10; b and c, ×20).

The findings of the histopathologic evaluation for each group are shown in Fig. 1. The histological grading values of testicular tissue damage observed in all preconditioning groups were less than group 1 (p < .05) with no grade 3 damage, which was observed in group 1. Examples of the different grades of testicular histopathological injuries in different groups could be found in Fig. 2 in which different magnifications were used for demonstrating cellular details better.

## Discussion

The protective effects of ischemic preconditioning, a phenomenon by which a traumatic or stressful stimulus confers protection against subsequent injury, have been well documented in many organs other than testis such as heart, brain, skeletal muscle, lung, liver, intestine, kidney, retina and endothelial cells [5-9]. However, Ceylan et al. claimed in a recent study that there are no protective effects of ischemic preconditioning on rat testicular ischemic injury [10]. These investigators studied different durations of early phase ischemic preconditioning periods (5, 10 and 3 times 10 min transient ischemia + 10 min reperfusion) followed by a 90-min ischemia. But it is difficult to be sure that IP has no protective effect on ischemic testicular tissue by only this one study by Ceylan et al. In order to further investigate this issue, the effects of ischemic preconditioning on biochemical and histological changes were examined in the present study. The purpose was to determine if multiple combinations of ischemic preconditioning have a protective effect on testicular tissue damage following 180 minutes of main ischemia, which is a period of ischemia longer than that used in the previous study [10].

It has been shown that ischemic preconditioning is a biphasic phenomenon, with an early and late phase of protection. There is increasing evidence that the ischemic stres hormones adenosine and norepinephrine are involved in the early phase of preconditioning, acting through the activation of protein kinase C [15-18]. Cellular stressors activate protein kinase via G-protein-coupled receptor binding and membrane phospholipase activation. The signal transduction cascade of preconditioning involves activation of protein kinase C, protein tyrosine kinase, and mitogen-activated protein kinase. Miura et al. [19] and Sandhu et al. [20] found that protein kinase C inhibitors could attenuate the effects of ischemic preconditioning induced by one cycle, but not repetitive cycles in the heart. Similarly, another study demonstrated that a radical scavenger N-2-mercaptopropionylglycine abolished protection afforded by a single cycle but not four cycles of preconditioning in rabbit hearts [21]. These data suggest that repetitive ischemic preconditioning may also activate additional mechanisms other than antioxidant systems. That is why we added experimental group 5, 6, and 7 in which testicles had repetitive cycles of preconditioning just before main ischemia.

Lipid peroxidation stimulated by free radical formation is one of the most important mechanisms involved in cellular damage and death. Free oxygen radicals induce peroxidation of unsaturated fatty acids of the cell membrane, if severe enough, destroying membrane integrity. MDA level measurements are widely used as an indicator of lipid peroxidation [22-24]. Several studies have shown that free radicals are generated and MDA levels are elevated during testicular ischemia [10,25]. The effect of ischemic preconditioning on the ischemia-induced increase in tissue MDA levels was studied in isolated guinea pig lungs [22], and in rat testis [10]. In guinea-pig lungs, ischemic preconditioning was found to prevent MDA increase, whereas no change could have been observed in rat testis. On the other hand, Unsal et al and Yang et al [26,27] have reported that free radical scavengers rescued testicular function after an experimental torsion of 2 hours, and these finding can be accepted as evidence for the presence of the negative effect of free radicals in torsion-induced ischemia. Interestingly, in the present study in which the duration of ischemia was doubled (180 min) compared to the study of Ceylan et al [10], both single and triple applications of 10 min (group 2, group 5) ischemic preconditioning significantly reduced MDA increase in response to ischemia in rat testicular tissue.

Ozokutan et al suggested that NO plays an important role in damaging the testis with ischemia-reperfusion [28]. Although inhibition of NO synthesis with L-NMMA, a competitive inhibitor of NO synthase, significantly improves ischemia-reperfusion injury in testes, enhancing NO production by providing excess L-arginine increases such damage. The role of NO in ischemic preconditioning was also suggested by Cho et al in ischemic preconditioned mice [29]. We also measured NO levels to determine biochemical damage in testicular tissue as an additional parameter to MDA. In the present study, NO levels were numerically decreased in all experimental groups compared to group 1 but this was statistically significant in only group 5.

There was no certain correlation between biochemical test results and histological observations in our study. In contrast to biochemical test results, histological examination of the testes showed significant improvement in all experimental groups subjected to ischemic preconditioning compared to controls. While grade 3 injury was not observed in any preconditioned group, it was seen in group 1. All our histopathological findings support that ischemic preconditioning has a protective effect on testicular tissue. The reason why biochemical test results could not reach the level of statistical significance in all groups unlike histological observations might be related to the small number of rats used in each group. Therefore, further studies with an increased number of rats and different preconditioning protocols are needed to confirm our suggestion that ischemic preconditioning has a protective effect on testicular ischemic tissue injury.

We conclude that our results suggest that ischemic preconditioning performed just before main ischemia provides tissue protection in testicular tissue.

## List of abbreviations used

malondialdehyde (MDA), nitric oxide (NO)

## Competing interests

The author(s) declare that they have no competing interests.

## Authors' contributions

TS and UKO participated in the design of the study, carried out animal model and drafted the manuscript. HC carried out the pathological examination and participated in the sequence alignment. FIT and SSI carried out the biochemical examination. All authors read and approved the final manuscript.
